# Synthesis of the extracellular domain of GLP-1R by chemical and biotechnological approaches†

**DOI:** 10.1039/d2ra02784d

**Published:** 2022-08-26

**Authors:** János Szolomajer, Pál Stráner, Zoltán Kele, Gábor K. Tóth, András Perczel

**Affiliations:** Department of Medical Chemistry, Albert Szent-Györgyi Medical School, University of Szeged H-6720 Szeged Hungary toth.gabor@med.u-szeged.hu; MTA-ELTE Protein Model. Res. Group and Laboratory of Structural Chemistry and Biology Pázmány P. stny. 1/A 1117 Budapest Hungary perczel@chem.elte.hu; Laboratory of Structural Chemistry and Biology, Institute of Chemistry, ELTE Eötvös Loránd University Pázmány P. stny. 1/A 1117 Budapest Hungary; MTA-SZTE Biomimetic Research Group, University of Szeged H-6720 Szeged Hungary

## Abstract

The extracellular domain of the glucagon-like peptide-1 receptor, GLP-1R, is responsible for the binding of GLP-1, and a handful of additional agonists (such as exenatide, lixisenatide, and liraglutide) used daily for treating type II diabetes mellitus. Lead discovery and optimization, however, require binding studies, which, in turn, necessitate the total synthesis of GLP-1R, comprising 108 residues. A protein domain of 10–15 kDa size could be obtained either by expression in *E. coli* or by ligating solid-phase peptide synthesis (SPPS)-made fragments. However, direct overexpression fails to give a properly folded protein, as GLP-1R forms an inclusion body, which fails to refold due to improper disulfide pairing. Several bacterial strains, constructs, and fusion partners were probed and it was found that only co-expression with MBP gave a 3D-fold allowing the native disulfide bond pattern formation. Some fusion partners can act as covalently linked or *in situ* chaperones for guiding the refolding of GLP-1R toward success. Therefore, the bottleneck to preparing GPCR extracellular domains is the correct pairing of the Cys residues. As a proof-of-concept model, nGLP1-R was made by SPPS to form the purified full-length polypeptide chain, subjected to self-guided or spontaneous Cys pairing. However, the formation of correct SS-pairs was lagging behind any protocol in use support, and the bottleneck of large-scale protein production relies on the risky step of proper refolding, which is sometimes possible only if a suitable fusion partner effectively helps and catalysis of the correct disulfide formation.

## Abstract 2

A 10–15 kDa, 3-disulfide-bridges containing protein domain is obtained by SPPS and is expressed in *E. coli*.

## Graphical abstract

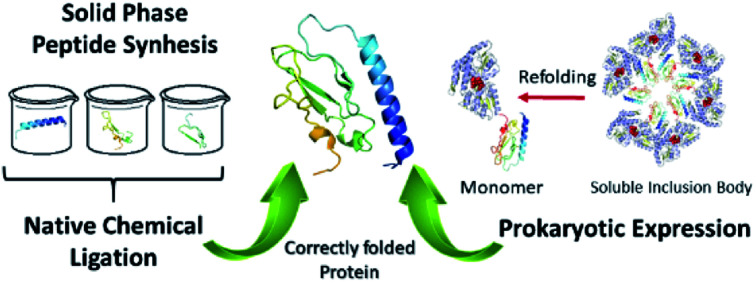

## Introduction

Increased insulin utilization to treat type II diabetes mellitus (DM) can lead to dysfunction of the pancreatic β-cells and their subsequent destruction, which can ultimately lead to a decrease and cessation of insulin production and secretion. Due to the decreased insulin concentration, the body's glucose homeostasis is disrupted, which can cause hyperglycemia and other serious complications (vasoconstriction, infarction, blindness, *etc.*). Current therapies use mainly externally administered insulin or sulfonylurea derivatives. The main disadvantage of these two approaches is that they continue working even after the restoration of optimum glucose levels, and thus their improper administration can lead to hypoglycemia.^1^ On the contrary, the advantage of GLP-1 receptor agonists, including exendin-4, is that their insulin production is stimulated only in the presence of elevated blood glucose levels and therefore, there is no need to fear hypoglycemia due to overdose.^2^ A comparative study of bacterial expression and/or the solid-phase peptide synthesis (SPPS) of shorter polypeptides and mini proteins 20–40 amino acids long related to GLP-1 was conducted.^3^ We highlight clear differences, such as the non-selective ^15^N-, ^13^C-isotope labeling that is more economical to do by expression and why SPPS can be faster and easier to be automated, especially using flow chemistry.^4^ The structural characterization of Trp-cage mini proteins revealed no difference in the strategy taken to make GLP-1 agonist-like polypeptides^5^ and the rational design of α-helix-stabilized exendin-4 analogues was successful.^6^

The GLP-1 receptor is a B-family G protein-coupled receptor with an extracellular domain of 100–130 amino acids that binds up to 27-residues-long endocrine peptide hormones. There are currently 31 X-ray and 4 NMR extracellular domain 3D structures (PDB) all of which are expressed in *E. coli*.^7,8^ The sequence identity of the extracellular domain of family B members of GPCRs is low. The important structural feature of the extracellular domain is the “complement control protein”-fold (CCP), an α–β–β/α architecture. Its central core consists of two antiparallel β-folds stabilized by 3 disulfide bridges and hydrophobic interactions. The N-terminus of the domain is formed by a longer α-helix linked by an SS-bridge to the first fold, thus forming a ligand-binding pocket. The sequence homology between the extracellular domains of family B GPCRs is surprisingly low, with essentially 6 Cys residues and just about a dozen amino acid identities.^9^

Three different protocols are described to express GLP-1R in *E. coli*. (i) First the “direct” production of the extracellular domain, mostly with an N-terminal His-tag, by fermentation. In each case, the target protein was isolated from inclusion bodies, followed by a refolding process.^10–13^ (ii) Second, the expression of GLP-1R with TrxA fusion protein in Origami cells, subsequently cleaved with thrombin.^10^ However, in this case, the expressed nPTHR construct was accumulated in inclusion bodies, which after refolding was degraded during thrombin cleavage. Presumably, the degradation occurred due to the high concentration of misfolded protein.^14–16^ (iii) The third method was used to make nPTHR by fusing the construct to MBP and co-expressing with DsbC in Origami B cells.^17^ Following the expression, the fusion protein was refolded in a GSH/GSSG redox system in the presence of DsbC. Note that the target protein was not separated from the MBP and occasionally, DsbC isomerase was not used during refolding.^17^

The direct expression of the extracellular domain of the GLP-1R was published in 2002 by Bazarsuren *et al.*^10^ and by Schröder-Tittmann.^11^ These methods seem hardly feasible in the absence of a large-scale fermentor and refolding reactor. Nevertheless, we completed the expression in a conventional incubator shaker. However, our first trials following the original protocol were unsuccessful. This was presumably due to the efficiency of the fermentation,^10^ in which presumably ∼700 g of cell pellets and ∼10 g of inclusion bodies were isolated from liters of the medium. Presumably from the cell pellets, ∼10 g of inclusion bodies were isolated, in contrast: a conventional shake of 1 L of rich medium contains about 5 g of cell pellets, *i.e.* 70 mg of IB. During renaturation, a large amount of precipitation was observed due to the misfolding of the GLP-1R.

In summary, we failed to reproduce the soluble form of GLP-1R with native SS-bridge pairing when adapting the original protocol to conventional shaking culture. However, here we describe an MBP-fusion expression system we successfully used to purify the correctly folded GLP-1R from a conventional shaking bacterial culture.

## Aims

In the absence of a large volume fermenter and refolding reactor, as is true of most labs, we aimed to enhance the yield of the native GLP-1R expressed in *E. coli* using ordinary incubator shakers. Our concept was to increase the refolding efficiency from lower amounts by using (i) domain optimization in the case of direct expression; (ii) alternative bacterial strains, and (iii) chaperone-like fusion proteins (Table 1). We show the comparative analysis of these approaches with the synthesized full-length, 108-residues-long GLP-1R domain.

**Table 1:** GLP-1R constructs probed

Construct type	Fusion partner/cleaving enzyme	GLP-1R variant
GLP-1R	N-term. His-tag & thrombin	24–145
R132	—	24–132
MBPthr24-132	N-term. MBP &TEV	24–132
nsDR132	N-term. DsbC with signal sequence & His-tag & thrombin	24–132
sDR132	N-term. DsbC without signal sequence & His-tag & thrombin	24–132
GST132	N-term. GST & thrombin	24–132
Ubq132	N-term. ubiquitin	24–132
Trx 132	N-term. His-tag & TrxA & thrombin	24–132

## Results and discussion

### Protein expression, purification, and refolding

**Fig. 1:**
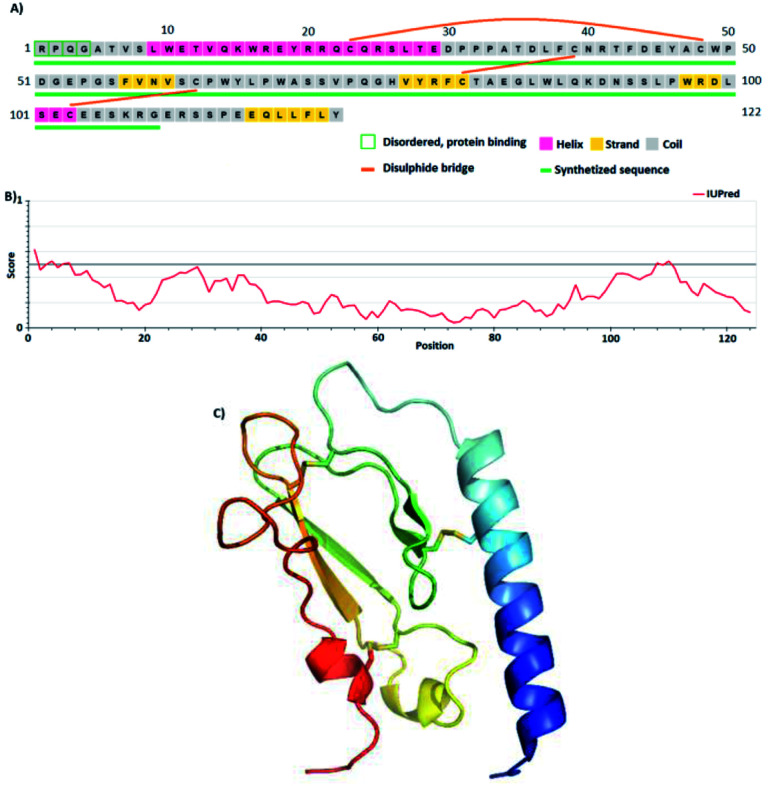
(A) Amino acid sequence and secondary structure properties of GLP-1R. The expressed and synthesized protein is underlined in green. (B) IDP/fold propensity as a function of the primary sequence of nGLP-1R, by IUPRED signals a structured but highly dynamic protein fold. (C) X-ray-determined 3D structure of GLP-1R (PDB 3C5T) without a ligand attached to it.

**Fig. 2:**
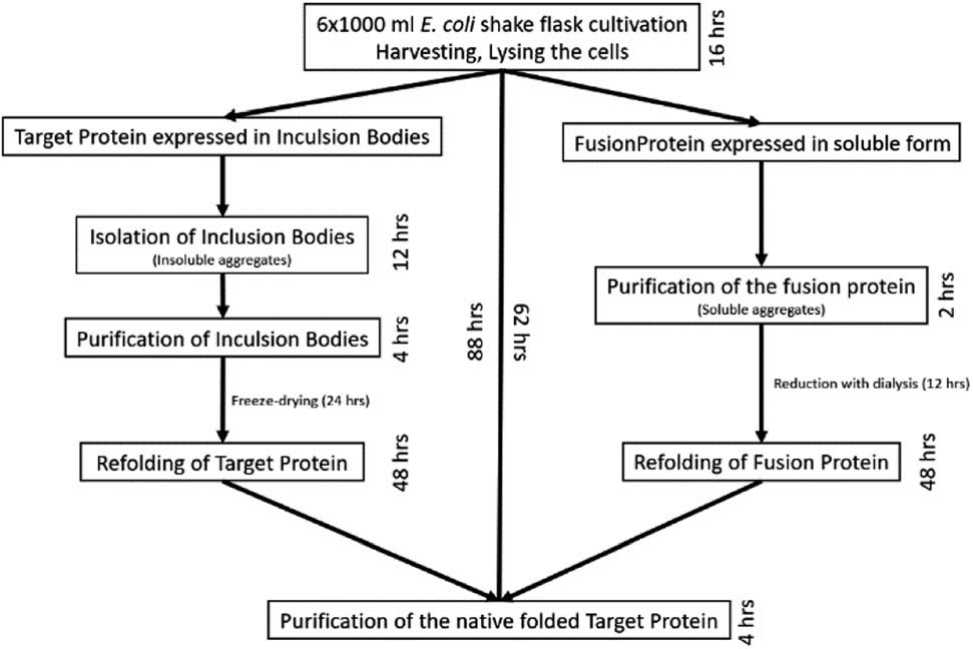
Expression and purification scheme of GLP-1R from the inclusion bodies and soluble forms.

The successfully crystallized and X-ray determined (PDB 3C5T) GLP-1R was made in *E. coli*, forming inclusion bodies (IBs). However, protein solubilization and refolding from IBs is a very inefficient, time-consuming, and costly process.^8,9^ Therefore, to enhance the yield and efficiency of a direct and soluble expression, we modified the original protocol as well as the expressed GLP-1R DNA-construct as follows (Fig. 1 and 2).

Concerning the original 1–147, (i) the N-terminal 1–23 signal sequence of the GLP-1R was cut off, resulting in GLP-1R (24–147) (Table 1); (ii) furthermore, we further reduced and optimized the protein size at the C-terminus as well. The crystal structure of the GLP-1R contained 28–131 residues only, so the C-terminal 132–147 region must have been indeed flexible at least in the crystal. Therefore, *via* its enhanced internal dynamics, this bit might disturb protein refolding. In addition, in the original construct, the C-terminal hydrophobic -^141^LLFLY- sequence can probably form an association with the membrane or embed into it. Therefore, we made a truncated GLP-1R variant, comprising 24–132 residues only GLP-1R^24–132^ abbreviated as R132 (Fig. 1B and C); (iii) additionally, we removed the His-tag from the N-terminal of the GLP-1R, as the purification of the IBs could be accomplished by RP-HPLC, resulting in a protein “ready” for MS analysis and for exact concentration adjustment for a refolding reaction. On the other hand, the reduction of IBs and the conditions of the subsequent renaturation process (DTT, EDTA, Arg) did not allow Ni-IMAC purification by the His-tag, which necessitated the introduction of an additional dialysis step. After the refolding reaction, the purification of the folded protein could be made by IEX. Another aspect of this modification was in line with the reported corticotrophin receptor purification protocol, where it was found that the His-tag interferes with the formation of the proper SS-bridge pattern.^15^

The refolding reactions were performed by diluting the isolated IBs (unfolded GLP-1R in 6 M Gua HCl, 100 mM DTT) to a large volume in redox buffer as detailed by the original protocol.^10^ Interestingly, we did not find any benefit of varying the concentration of l-Arg as outlined in the original GLP-1R protocol. Following renaturation, dialysis was required as the presence of l-Arg interferes with the downstream chromatography (Q-IEX, RP-HPLC). After dialysis, nearly 90% of the target protein was precipitated. The folded GLP-1R was purified from the soluble phase using Q-IEX and RP-HPLC afterwards. With this method, 0.05 mg truncated GLP-1R was purified from 70 mg IBs. We analyzed this final product using mass spectrometry and by disulfide bridge pattern analysis. Besides the proper molecular ion, however, the MS data revealed two additional proteins, as the GLP-1R was degraded during the renaturation processes, which raises the possibility of protein instability and course, reducing the overall production yield.

We searched for new production pathways due to the low and uncertain yield. The focus was to avoid IBs formation of the target protein as these would be difficult and cumbersome to handle (Fig. 2). The major problem with expressing multiple disulfide bridges containing proteins in *E. coli* is the reductive environment of the cytoplasm. In contrast, the cytoplasms of Origami B and Shuffle strains are oxidative due to their mutations,^18,19^ therefore the formation of disulfide bridges in the cytoplasm was pursued. In addition, Shuffle (DE3) cells contain a cytoplasmic DsbC, which enhances the correct formation of the disulfide bridge pattern.^19^

To express proteins containing disulfide bridges in *E. coli*, these strains were therefore considered appropriate. However, in the case of “not-so” globular/partially disordered regions containing protein (Fig. 1), like GLP-1R, the direct expression of the target protein in these oxidative strains does not work. However, “problematic cases” can be absolved by using a suitable protein fusion tag at the N-terminal, like that of thioredoxin, DsbC, MBP, GST, Ubiquitin, SUMO, *etc.*^20^ Along this line, we constructed a pET-based DNA-vector family, in which the target protein can be cloned to the C-terminal part of the fusion partner with the same restriction site, frame, and position. We tried to express these DNA constructs using different expression parameters (induction time/inductor concentration/temperature, *etc.*) and different expression strains (Table 2). Summarizing these constructs and experiments, protein production could be divided into “successful” and “unsuccessful” cases. In all cases judged “unsuccessful”, the fusion protein formed an inclusion body. On the other hand, in the “successful” cases the fusion protein remained in the cytoplasm in soluble form, so the first purification step was performed directly after the cell lysis. However, the latter cases did not necessarily mean that the corresponding disulfide bridge pattern of the GLP-1R target protein was formed in the cytoplasm.

**Table 2:** Summary of the expression conditions

*E. coli* strain		BL-21 (DE3)				
		Shuffle (DE3)				
		Origami B (DE3)				
Induction	Temp.	16 °C–18 °C–22 °C–26 °C–30 °C–37 °C				
	Inducer conc. (IPTG)	0.05 mM 0.1 mM 0.2 mM 0.5 mM 1.0 mM				
	Time (h)	3–6–12–18				
Fusion protein	IBs	noTag	Ubq	TrxA	GST	SUMO
	Cytoplasm	MBP		DsbC		

The TrxA fusion expression protocol turned out to be “unsuccessful”. In every used expression strain, the fusion protein formed IBs. Therefore, thioredoxin did not exert its published chaperone activity in the cytoplasm of the Shuffle (DE3) and Origami B (DE3) cells.^17–19^ Interestingly, during the subsequent refolding step, though the fusion protein remained in solution, after thrombin cleavage, the GLP-1R was not detected by SDS-PAGE. This result indicated that the GLP-1R was not perfectly folded,^16^ and thus, thrombin protease cleaved it. Similarly, the use of ubiquitin, SUMO, and GST labels was similarly unsuccessful.

The soluble, cytoplasmic productions of DsbC- and MBP-fused GLP-1Rs were successful, but only using the BL21 and Shuffle strains. After harvesting these cells, the fusion protein was in the cytoplasmic phase, so the first chromatographic purification step from the supernatant fraction was completed. This shows that the use of DsbC and MBP as fusion partners brings the target protein into the solution phase, which simplified the forthcoming purification steps, but did not presuppose the formation of a proper disulfide bridge pattern, as shown in the case of thioredoxin.

**Fig. 3:**
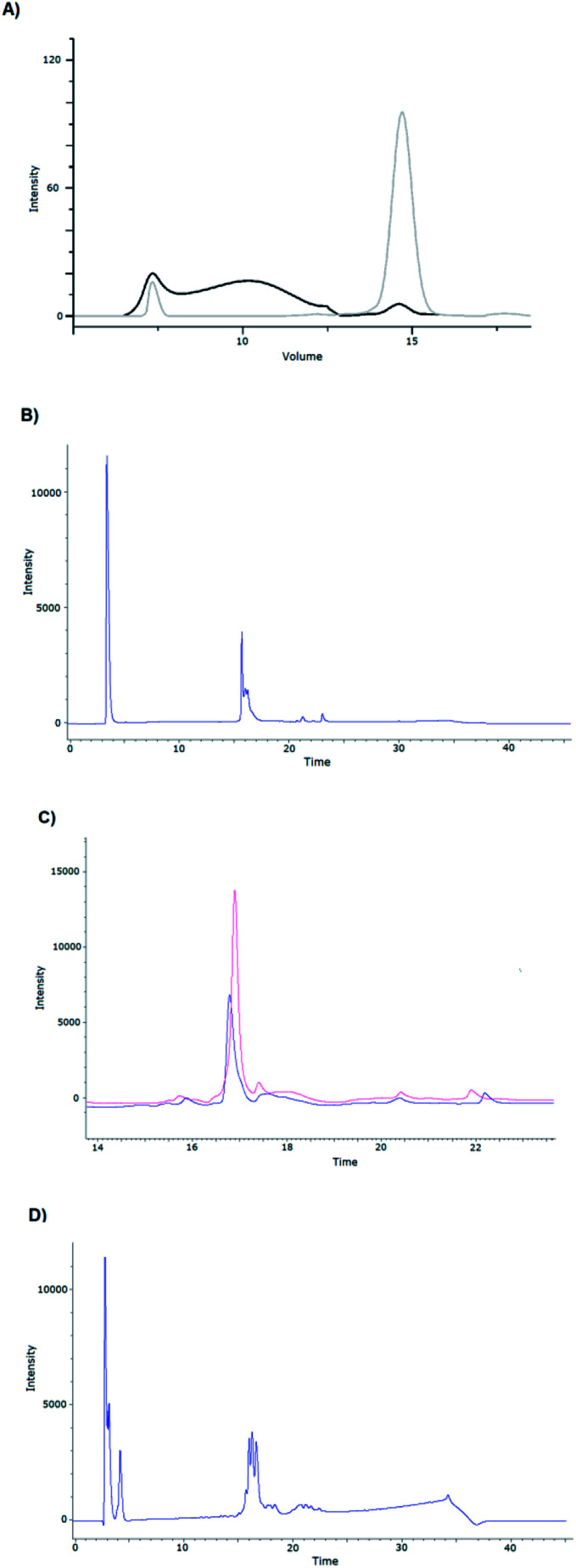
(A) Size-exclusion chromatogram of the MBP-fused R132 construct (Table 1), following Ni-IMAC chromatography without refolding (black line) and after refolding (gray line). The “extra” renaturing step introduced decreased the oligomeric form (7.5 ml) and increased the monomeric form (14.8 ml). Analytical RP-HPLC chromatograms of (B) the refolded R132; (C) MBP-R132 before (blue line) and after refolding (red line) and (D) that of the DsbC-R132 construct after refolding.

When we examined the role of the three bacterial strains, it was surprising that the MBP- and DsbC-fused GLP-1R did not form IBs in the reductive cytoplasm of BL21 (DE3), suggesting that MBP and DsbC may have a solubilizing or chaperone effect. After the first purification step (Ni-IMAC), the eluted fractions were analyzed by size-exclusion chromatography, and a large oligomeric form of the receptor was typically detected. Therefore, even though the latter two fusion partners could solubilize the GLP-1R, soluble aggregates or solvated IBs were formed (Fig. 3). Therefore, we introduced a renaturing “extra” step following the first purification phase, and indeed, the subsequent size-exclusion chromatography showed a decrease in the oligomeric form and an increase in the monomeric form (Fig. 3A).

The need for the extra renaturing step was also supported by the analytical RP-HPLC chromatograms. Analysis of the R132, MBP-R132, and DsbC-R132 constructs revealed that the elution profiles did change for the better (Fig. 3B and C).

Previously, we have shown that co-expressed DsbC enhances the refolding efficiency.^17,18^ In our experiments, the co-expressed isomerase was found after the first Ni-IMAC purification steps in all the eluted fractions, *i.e.*, it binds unspecific to the matrix or this effect is caused by the activity of DsbC isomerase. It binds to its substrate (in this case, the target GLP-1R protein) as an enzyme does after proteolytic cleavage. However, after empty harvesting of the Shuffle cells, genomically encoded DsbC could not be eluted by Ni-IMAC. For this reason, renaturation was performed in a GSH/GSSG redox environment without DsbC. After refolding, the DsbC-fused GLP-1R was investigated by analytical RP-HPLC. Several intense peaks appeared on the chromatogram, which were identified as belonging to the fusion protein by SDS-PAGE. Presumably, this was caused by the formation of the proteins with different disulfide bridge patterns.

**Fig. 4:**
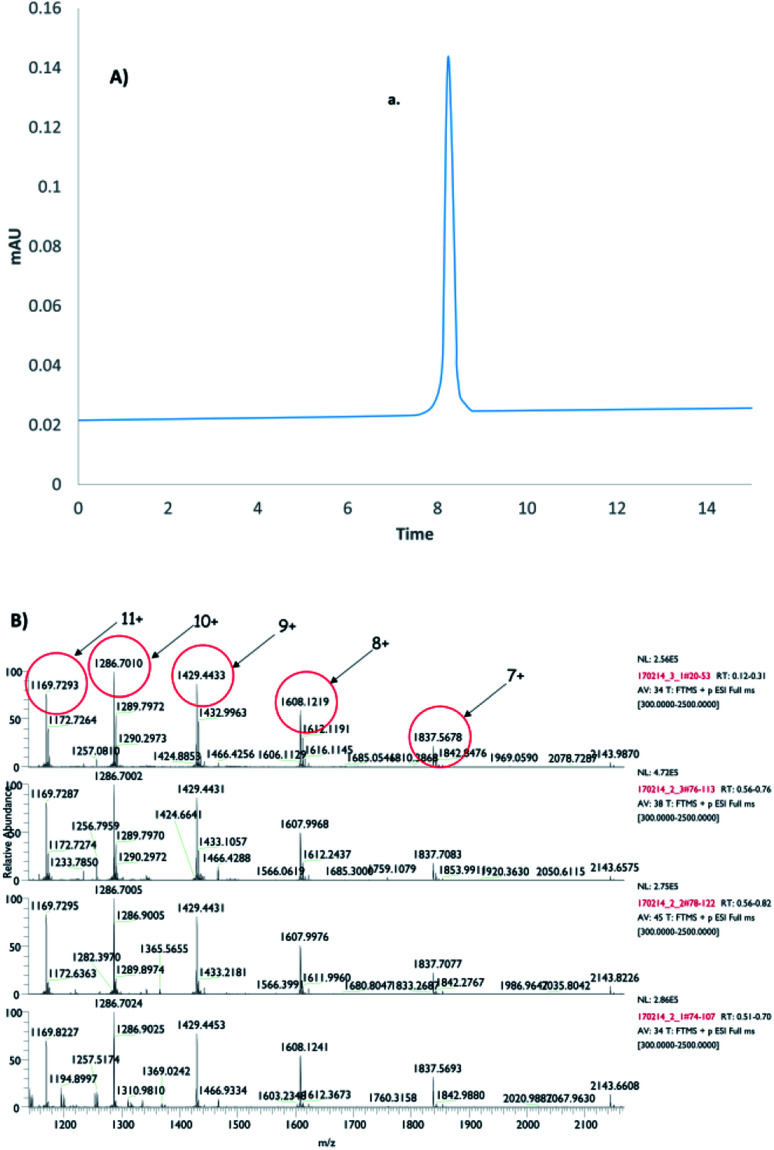
(A) Analytical RP-HPLC chromatogram of the expressed, oxidized GLP-1R having the native GPCR 3D-fold (peak a.) including the proper SS-bond pairings (Fig. 1). (B) MS spectrum of GLP-1R: MW_calculated_: 12857.12 and MW_measured_: 12857.01.

The MBP-fused variant was purified after the refolding by amylose affinity chromatography, followed by proteolytic cleavage. The GLP-1R was purified by reverse Ni-affinity chromatography. Finally, the “pass-through” fraction contained the coveted product, *i.e.,* the properly folded GLP-1R. This was purified by reverse-phase HPLC and subjected to mass spectrometric analysis (Fig. 4). The MS unquestionably proved the formation of disulfide bonds (Mw_calculated_: 12857.12, Mw_measured_: 12857.01), and thus, the expected correct disulfide pattern was determined by enzymatic methods followed by UPLC-MS analysis (Fig. 1). However, based on disulfide bridge pattern analysis, two other disulfide-bridge-patterned GLP-1Rs were also detected in the solution phase, indicating that the MBP-guided protein refolding was imperfect, as it did not result in a single product. The produced amount of the native disulfide patterned target protein was 0.5 mg made from 6 L of nutrient culture. Therefore, compared to the other expression yields, a subtle but significant increase was observed. In addition, the advantage of this method is undoubtedly its simplified, cost-effective, and easy-to-use protocol.

To demonstrate that in the absence of MBP-guided refolding the proper SS-pairing of GLP-1R is unlikely to be obtained in a reproducible and large-scale manner, the chemical synthesis of GLP-1R using SPPS and NCL was completed. Due to the length and the difficulty of the 108-amino-acid-containing GLP-1R protein sequence, the step-by-step manual synthesis of the protein would have been inefficient and time-consuming, so we decided to synthesize the GLP-1 receptor protein by a combination of manual and automated solid-phase peptide synthesis completed with native chemical ligation.^21–24^ The designed peptide fragments were fully compatible with the native chemical ligation procedure and were synthesized using a CEM® Liberty Blue microwave-assisted automated peptide synthesizer.

**Fig. 5:**
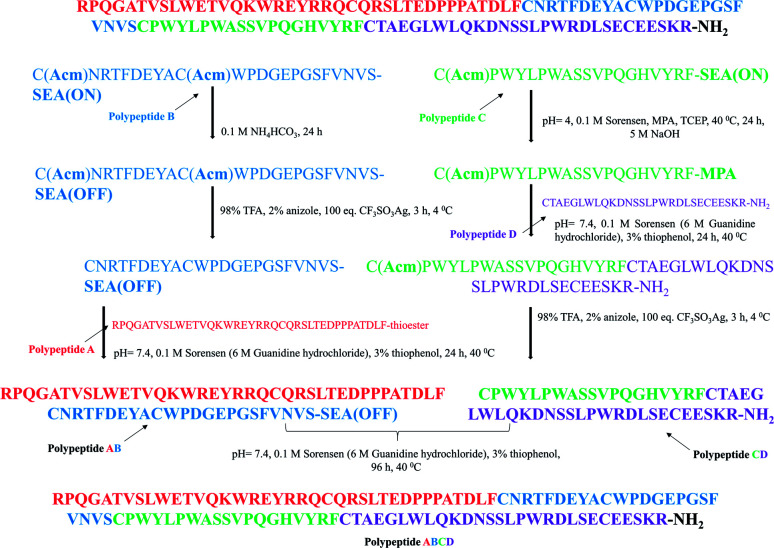
Key steps of the chemical synthesis of the GLP-1R protein made from the rational assembly of polypeptides A, B, C, and D *via* SEA.

The first polypeptide A thioester derivative was synthesized using manual SPPS and Boc chemistry. The C-terminal first amino acid, Phe (F), was coupled to the free sulfhydryl group of cysteine. Reaching full length, the thioester was detached from the resin using HF, and the crude polypeptide A was purified by C18 RP-HPLC. Polypeptide B was made on SEA resin,^23^ with the Cys residues Acm side chains protected. Exploiting the advantages of SEA chemistry, the crude SEA-(ON) polypeptide B was oxidized with ammonium hydrogen carbonate (0.1 M) to obtain the crude SEA-(OFF) peptide. Note that the SEA-(OFF) peptide B is unreactive at its C-terminal, which avoids the formation of ligation side products during chemical ligation.^24^ Before the chemical ligation of A to B, the Acm protecting groups were removed by Ag(OTf) in TFA/anisole (4 °C and 4 h) and the crude Acm-deprotected polypeptide B was purified by C18 RP-HPLC. The chemical ligation of the thioester of polypeptide A and SEA-(OFF) polypeptide B were ligated in a Sorensen buffer (pH 7.4) in the presence of 3% thiophenol (40 °C for 24 h) (Fig. 4 and 5) resulting in the 61-amino-acid-containing N-terminal fragment (polypeptide AB) of GLP-1R, purified by C18 RP-HPLC (Fig. 5).

**Fig. 6:**
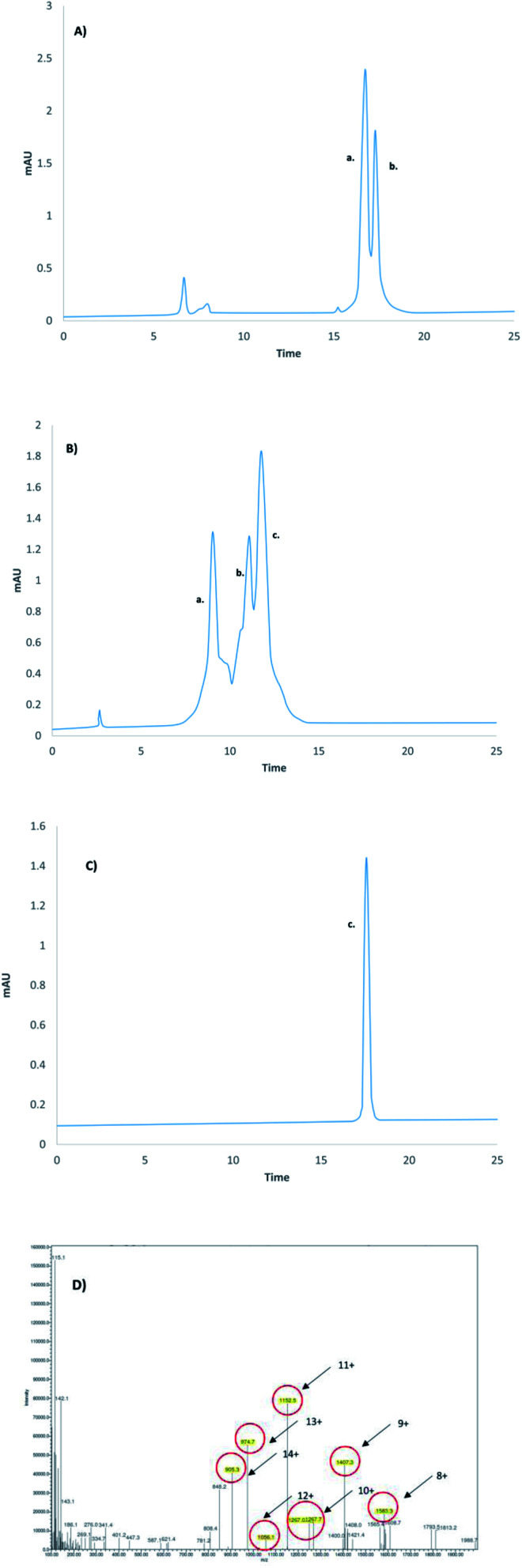
Chemical ligation of the 61-amino-acids-containing “N”-terminal SEA-(OFF) fragment (peak b.) and the 47-amino-acids-containing “C”-terminal peptide amide (peak a.) (A) at 0 min (5–80% B, 25 min, 1.2 ml min^−1^) and (B) after 96 h (38–58% B, 20 min, 1.2 ml min^−1^). (The formation of the 108-amino-acids-containing linear GLP-1R protein (peak c.) was detected.) (C) The analytical RP-HPLC chromatogram (5–80% B, 25 min, 1.2 ml min^−1^) of the pure linear GLP-1R protein was obtained by chemical ligation. (D) MS spectra of the pure linear GLP-1R. The highlighted peaks correspond to the MW of the linear GLP-1R. LC conditions: (A) 5–80% B, 25 min, 1.2 ml min^−1^, 220 nm, (B: 80% ACN); (B) 38–58% B, 20 min, 1.2 ml min^−1^, 220 nm (B: 80% ACN); (C) 5–80% B, 25 min, 1.2 ml min^−1^, 220 nm, (B: 80% ACN).

**Fig. 7:**
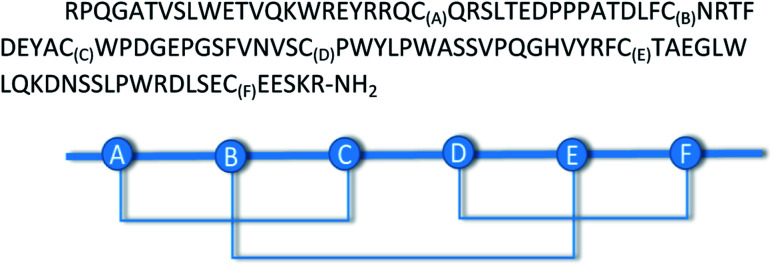
Native SS-bond topology of GLP-1R, C1–C3, C2–C5, and C4–C6 disulfides.

Polypeptide C was synthesized on SEA resin by applying Fmoc/*t*Bu chemistry and Acm protection for Cys residues. The active SEA-(ON) carboxyl-terminal of the crude peptide was converted into a more reactive MPA thioester in the presence of tris(2-carboxyethyl)phosphine (1000 eq.) in slightly basic media (0.1 M Sorensen buffer pH 7.4) at 40 °C for 24 h.^24^ The crude Acm-protected MPA thioester was purified by C18 RP-HPLC. The C-terminal part of the C-terminal fragment, polypeptide D, was made as the method described above for polypeptide C. The chemical ligation of the Acm-protected-MPA thioester of polypeptide C and D resulted in the 47-amino-acid-long C-terminal polypeptide CD (Fig. 5). Ligation was carried out in slightly basic media (0.1 M Sorensen buffer, pH 7.4) in the presence of thiophenol at 40 °C for 24 h (Fig. 6 and 7). The C-terminal fragment of GLP-1R was purified by C18 RP-HPLC. Cys (Acm) deprotection of the C-terminal fragment, polypeptide CD, was completed as described above (Ag(OTf) in TFA/anisole, 4 h at 4 °C) and then purified (by C18 RP-HPLC) (Fig. 5).

The chemical ligation of the 61-amino-acids-containing N-terminal SEA-(OFF) (polypeptide AB) and the 47-amino-acid-long C-terminal polypeptide CD resulted in the 108-amino-acid-long linear GLP-1R protein, called polypeptide ABCD (Fig. 6A and B). The chemical ligation of the N-terminal SEA-(OFF) and Acm-deprotected C-terminal fragments was carried out in slightly basic media (0.1 M Sorensen buffer, pH 7.4, 6 M guanidine hydrochloride) in the presence of thiophenol (3%) at 40 °C for 96 h.^24^ The crude 108-amino-acids-containing linear GLP-1R was purified by C18 RP-HPLC and analyzed by LC-MS (Fig. 6C and D). The primary sequence of the 108-amino-acid-long GLP-1R, polypeptide ABCD is as follows: RPQGATVSLWETVQKWREYRRQCQRSLTEDPPPATDLFCNRTFDEYACWPDGEPGSFVNVSCPWYLPWASSVPQGHVYRFCTAEGLWLQKDNSSLPWRDLSECEESKR-NH_2_

### Self-guided SS-pairing and GLP-1R folding

As the last step of the chemical or biotechnological synthesis, the final goal was to obtain the correct disulfide pattern between the thiol groups by the oxidation of the 108-mer linear protein domain. Because of the presence of six SH-groups (C_46(A)_, C_62(B)_, C_71(C)_, C_85(D)_, C_104(E)_, and C_125(F)_), the formation of three intramolecular SS-bonds was expected (Fig. 7). For molecules that, under native-like conditions, naturally fold in conformations ensuring an effective pairing of the right disulfide bridge pattern, chemically driven approaches to oxidize cysteine may not be required. To obtain the desired disulfide pattern, the 108-amino-acids-containing linear protein was oxidized under various reaction conditions (Table 3). During the oxidation, most parts of the dissolved protein precipitated. After dissolution, the products were investigated by RP-HPLC, and the disulfide topology was determined following enzymatic digestion and by MS–MS measurements. The earlier eluting fraction contained mainly the unnatural C1–C6, C2–C3, and C4–C5 disulfide patterns. The main peaks contained numerous disulfides (C1–C3*, C1–C4, C2–C3, C2–C4, C2–C5*, C3–C4, C3–C6, C4–C5, C4–C6*, C5–C6) including the desired natural ones, as marked with asterisks, but unfortunately, in an inseparable manner. Due to the high chromatographic similarity, even RP-HPLC columns having the best plate number could allow only a negligible resolution between the numerous disulfide isomers. In addition to the separation problems, the extremely wrong solubility of the oxidized protein made this approach unsuccessful.

**Table 3:** Unguided refolding and oxidation conditions were applied for the synthesized linear GLP-1R

Oxidation	Oxidation conditions
GLP-1R oxidation 1	pH 7.5, 0.1 M ammonium acetate buffer, 0.2 mg ml^−1^, air, 24–48 h
GLP-1R oxidation 2	pH 7.5, 0.1 M ammonium acetate buffer 1.55 mM GSH, 0.155 mM GSSG, (GSH : GSSG-10 : 1), 0.2 mg ml^−1^, 48–72 h
GLP-1R oxidation 3	pH 7.5, 0.1 M Sorensen buffer/6 M guanidine hydrochloride, 1.55 mM GSH, 0.155 mM GSSG, (GSH : GSSG-10 : 1), 0.2 mg ml^−1^, 48–72 h
GLP-1R oxidation 4	pH 7.5, 0.1 M Sorensen buffer/6 M guanidine hydrochloride, 10 mM GSSG, 2 mM GSH (GSSG : GSH-5 : 1), 0.4 mg ml^−1^, 48–72 h
GLP-1R oxidation 5	pH 7.5, 0.1 M ammonium acetate buffer (6 M guanidine hydrochloride), 0.2 mg ml^−1^, air, 24–48 h, cysteine
GLP-1R oxidation 6	pH 7.5, 0.1 M ammonium acetate buffer 1 mM GSH, 1 mM GSSG, (GSH : GSSG-1 : 1), 0.2 mg ml^−1^, 48–72 h
GLP-1R oxidation 7	pH 8, 0.1 M ammonium hydrogen carbonate/acetonitrile (1 : 1), 0.2 mg ml^−1^, 24–48 h, air
GLP-1R oxidation 8	pH 7.5, 0.1 M ammonium acetate buffer/acetonitrile (1 : 1) CLEAR-OX™ (4 eq.), 0.2 mg ml^−1^, 2–4 h, closed system
GLP-1R oxidation 9	pH 8.5, 0.1 M ammonium acetate buffer 0.2 mg ml^−1^, 24–48 h, air, cysteine, 10 °C
GLP-1R oxidation 10	pH 8.5, 0.1 M tris buffer 1 mM GSH, 5 mM GSSG, (GSH : GSSG-1 : 5), 1 mM EDTA, 500 mM l-arginine, 0.2 mg ml^−1^, 48–72 h, closed system
GLP-1R oxidation 11	pH 8.0, 0.05 M tris buffer 1 mM GSH, 1 mM GSSG, (GSH : GSSG-1 : 1), 1 mM EDTA, 1 M l-arginine, 150 mM NaCl, 0.2 mg ml^−1^, 48–72 h, closed system

## Conclusions

Protein domains of 10–15 kDa size could be obtained both by expression in *E. coli* and/or by ligating SPPS-made suitable fragments. Both strategies could lead to the desired product and the choice between the alternative methods seems to be optional. The presence of multiple disulfide bridges within the protein is usually not above the capability of these techniques. Interestingly, in the current GLP-1R case, unlike in its chemical synthesis, the biotechnological expression of the receptor protein resulted in the desired, correctly folded 3D structure only.

To the best of our knowledge, this seldom happens for intact domains and for complete globular proteins. However, for truncated/designed macromolecules and their fragments, such as GLP-1R, this scenario is to be expected more often and thus, suitable fusion partners (*e.g.,* MBP) can be used to get help for proper Cys pairing and cystine formation. Therefore, the application of carrier proteins/chaperones could be necessary, even if the chemical preparation and/or bacterial expression of the unfolded protein is successful. An additional advantage of using an appropriate fusion partner is that it could improve the solubilizing properties of the truncated protein and thus facilitate the kinetics of the proper refolding.

## Experimental section

### Chemical synthesis of the GLP-1R protein

Due to the difficulty and the length of the sequence, the synthesis of the 108-amino-acids-containing GLP-1 peptide receptor was carried out by native chemical ligation.^23,24^ The designed fragments compatible with native chemical ligation were synthesized using solid-phase peptide synthesis (SPPS) with a Fmoc/*t*Bu strategy applying a CEM^®^ microwave-assisted fully automated peptide synthesizer.

The synthesis of RPQGATVSLWETVQKWREYRRQCQRSLTED-PPPATDLF-thioester (polypeptide A) was carried out using manual solid-phase peptide synthesis applying Boc chemistry. First, a Fmoc-Cys(Trt)-OH (4 eq.) was attached to the MBHA (0.6 mmol g^−1^) resin using *N*,*N*′-dicyclohexylcarbodiimide (DCC, 4 eq.), and 1-hydroxy benzotriazole (HOBT, 4 eq.) coupling. After the Fmoc deprotection (20% piperidine/DMF), the obtained free amino group was acetylated (30% acetic anhydride/dichloromethane). The trityl group of the cysteine was removed by treatment with trifluoroacetic acid (TFA). The first amino acid was attached to the free sulfhydryl group of cysteine by applying DCC/HOBT (4 eq.) double coupling in the presence of 4-(dimethylamino)pyridine (DMAP, 0.4 eq.) (yield after HPLC purification: 25%).

The synthesis of CNRTFDEYACWPDGEPGSFVNVS-SEA(OFF) (polypeptide B) was completed by using an SPPS/CEM^®^ fully automated microwave-assisted peptide synthesizer, applying Fmoc/*t*Bu chemistry using SEA resin (0.13 mmol g^−1^) and Acm side-chain protection for the Cys residues. The crude SEA-(ON) peptide was oxidized using 0.1 M NH_4_HCO_3_ to obtain the crude SEA-(OFF) peptide. The Acm side-chain protection was removed by using Ag(OTf) (50 eq.) in TFA (10 mg ml^−1^) in the presence of anisole at 4 °C for 4 h (yield after purification: 15%). The chemical ligation of peptide thioester (Polypeptide A) and SEA-(OFF) peptide (Polypeptide B) was carried out in the presence of thiophenol (3%) in 0.1 M Sorensen buffer, pH 7.4 (6 M guanidine hydrochloride), at 40 °C for 24 h (overall yield after purification: 21%).

The synthesis of C(Acm)PWYLPWASSVPQGHVYRF-MPA (polypeptide C) was made by using an SPPS/CEM^®^ fully automated microwave-assisted peptide synthesizer applying Fmoc/*t*Bu chemistry, using SEA resin (0.13 mmol g^−1^). The side chain of the N-terminal cysteine was protected with the Acm protecting group. The crude Acm-protected SEA-(ON) peptide was converted into Acm-protected peptide-MPA thioester by using 3-mercaptopropionic acid (5 v/v%), in the presence of tris(2-carboxyethyl)phosphine hydrochloride (TCEP·HCl) (100 eq.) in 0.1 M Sorensen buffer (6 M Guanidine hydrochloride) at 40 °C, pH 4 for 24 h (yield after HPLC purification: 20%).

The synthesis of CTAEGLWLQKDNSSLPWRDLSECEESKR-NH_2_ (polypeptide D) was done by using an SPPS/CEM^®^ fully automated microwave-assisted peptide synthesizer, applying Fmoc/*t*Bu chemistry (yield: 30%).

The chemical ligation of the Acm-protected peptide-MPA thioester (polypeptide C) and the peptide amide (polypeptide D) was carried out in the presence of thiophenol (3%) in 0.1 M Sorensen buffer pH 7.4 (6 M guanidine hydrochloride), at 40 °C for 24 h (yield: 41%). Acm protection of the “C”-terminal peptide was removed by Ag(OTf) (50 eq.) in TFA (10 mg ml^−1^) in the presence of anisole at 4 °C for 4 h (overall yield: 22%). The chemical ligation of the N-terminal SEA-(OFF) peptide and the Acm-deprotected “C”-terminal peptide amide was carried out in the presence of thiophenol (3%) in 0.1 M Sorensen buffer, pH 7.4, (6 M guanidine hydrochloride), 0.2 M TCEP·HCl at 40 °C for 96 h (yield after purification: 19%).

### Oxidation of GLP-1R made by chemical and recombinant synthesis and identification of the SS-bridges by MS

To obtain the desired disulfide bridges, the purified 108-amino-acids-containing linear GLP-1R peptide obtained by native chemical ligation and the protein obtained by recombinant synthesis were oxidized using various oxidation conditions (see Table 3 in the Results section). Because of the presence of 6 cysteine residues (C_23(A)_, C_39(B)_, C_48(C)_, C_62(D)_, C_81(E)_, and C_103(F)_), the formation of three disulfide bonds was expected. For disulfide-bridge identification of the protein (GLP1), an enzymatic digestion method combined with mass spectrometry was used. Based on the sequence of the protein, the method was planned to produce a mixture of peptide fragments containing only one disulfide bond. Based on the sequence of the protein, a mixture of two enzymes (trypsin and chymotrypsin) was found to be a good settling. Fragments linked together through disulfide bridges were separated and analyzed by capillary reverse-phase UPLC coupled to the mass spectrometer. These peptides could be identified based on their unique masses and tandem mass spectrometric fragments. For searching for possible linked fragments, the MS-Bridge software was used (https://prospector.ucsf.edu/prospector/mshome.htm).

### DNA constructions

For direct expression, the GLP-1R domain (R132) was ligated between the NdeI and BamHI sites of the pET-32b vector. Between the NdeI and BamHI restriction site of the pET-32b vector, the cDNA of each fusion protein was ligated with an N-terminal His-tag and a C-terminal thrombin cleavage site.

### Expression, purification, and refolding of the IBs of GLP-1R

The expression targeted to IBs formation (direct expression) was performed in 2YT media, at 37 °C and with 180 rpm shaking. At OD600 = 1, the expression was induced with 1 mM IPTG for 5 h. The expression of DsbC- and MBP-fused GLP-1R was performed at 2YT and 180 rpm. The expression was induced with 0.2 mM IPTG for 12 h at 18 °C.

After the cell lysis, the cytoplasmic fraction was removed by centrifugation. The IBs-containing pellet was washed by NaPi buffer 3 times. After the last centrifugation step, the pellet was solvated with 20 ml 6 M guanidine hydrochloride and 50 mM DTT, and a 12 h long incubation was performed at 37 °C. The pellet was removed by centrifugation, and the solvated IBs were purified by C4 RP-HPLC. The eluted fraction was lyophilized and solvated by 4 M Gua HCl at pH 8.5 at 1 mg ml^−1^ concentration and the refolding reaction was performed: a small amount was dosed to the refolding buffer (50 mM Tris, 150 mM NaCl, 10 mM GSH, 1 mM GSSG, 10 mM EDTA pH 8.5) up to the 20 μg ml^−1^ fusion protein concentration at 18–20 °C for 48 h, with mixing at 250 rpm with a magnetic stirrer. The pellet was then removed by centrifugation, and buffer exchange was performed by dialysis (14000 rpm, 4 °C, 30 min) (50 mM Tris, 50 mM NaCl). The eluted fraction was purified by C4 RP-HPLC.

### Expression, purification, and refolding of MBP- and DsbC-fused GLP-1R

**Fig. 8:**
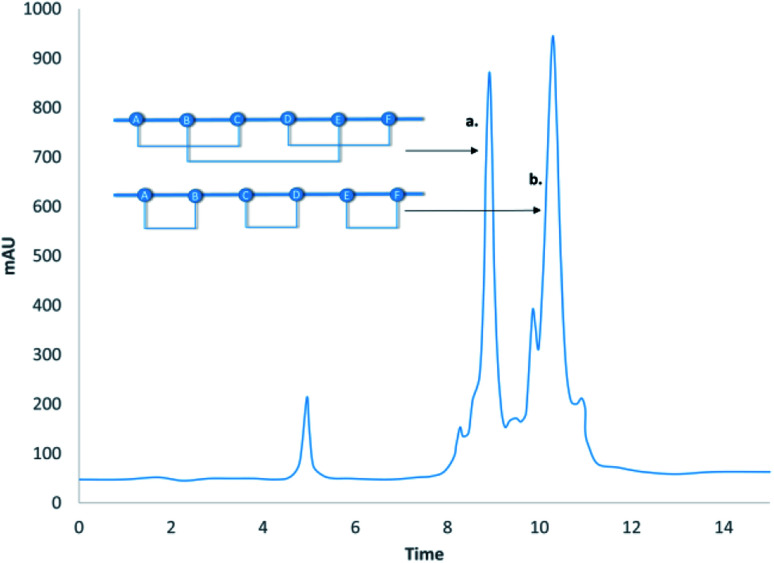
Oxidized forms of GLP-1R. Though the MBP-guided protein refolding process gave alternative S–S bond topologies of GLP-1R among the isolated proteins (peak a. and peak b.), the native fold (peak a.) could be identified.

After the cell lysis, the centrifuged cytoplasmic fraction of MBP- and DsbC-fused GLP-1R was purified by Ni-IMAC chromatography according to the manufacturer's protocol. A dialysis step was performed to remove the imidazole and to reduce the protein (50 mM Tris HCl, 150 mM NaCl, 5 mM DTT, pH 8.5). After the A280 concentration measurement, the refolding reaction was performed: a small amount was dosed to the refolding buffer (50 mM Tris, 50 mM NaCl, 10 mM GSH, 1 mM GSSG, 10 mM EDTA pH 8.5) up to the 20 μg ml^−1^ fusion protein concentration at 18–20 °C for 48 h, with mixing at 250 rpm with a magnetic stirrer. The pellet was then removed by centrifugation, and buffer exchange was performed by dialysis (14000 rpm, 4 °C, 30 min) (50 mM Tris, 50 mM NaCl). The fusion protein was purified and concentrated by Q-IEX chromatography. The eluted fraction was immediately cleaved with thrombin. After the incubation time, a second Ni-IMAC was performed, and the target GLP-1R passed through the column. This fraction was further purified by C4 RP-HPLC, which led to two major products (see Fig. 8, peaks 1 and 3) having the correct molecular mass. According to the mass spectrometrical investigations combined with enzymatic digestion, the disulfide patterns of the two isolated proteins proved to be C1–C3, C2–C5, and C4–C6, peak 3, (the natural one), and C1–C2, C3–C4, and C5–C6, peak 1, an unnatural isomer.

## Abbreviations

GLP-1: Glucagon-like peptide-1
GLP-1R: Extracellular domain of the glucagon-like peptide-1 receptor
GPCR: G-protein coupled receptor
SPPS: Solid-phase peptide synthesis
NCL: Native chemical ligation
SEA^on^: Bis(2-sulfanylethyl)amido group
MPAA: Mercaptophenylacetic acid
SEA^off^: The oxidized, cyclic disulfide derivative of SEA^on^
MBP: Maltose Binding Protein
DsbC: Disulfide bridge isomerase C
GST: Glutathione-S transferase
SUMO: Small ubiquitin-like modifier
TrxA: ThioredoxinA
TEV: Tobacco etch virus nuclear-inclusion-a endopeptidase
IMAC: Immobilized metal chelate affinity chromatography
Q-IEX: Quaternary anion ion exchange chromatography
GSH: Reduced glutathione
GSSG: Oxidized glutathione

## Author contributions

Conceptualization, A. P., and G. K. T.; methodology, P. S.; J. S.; Z. K.; writing-original draft preparation, J. S.; P. S.; writing-review and editing, G. K. T.; funding acquisition, A. P., and G. K. T. All authors have read and agreed to the published version of the manuscript.

## Conflicts of interest

There are no conflicts to declare.

## Supplementary Material

## References

[cit1] Mann K. V., Raskin P. (2014). Diabetes, Metab. Syndr. Obes.: Targets Ther..

[cit2] Meloni A. R., DeYoung M. B., Lowe C., Parkes D. G. (2013). Diabetes, Obes. Metab..

[cit3] Straner P., Taricska N., Szabo M., Toth G. K., Perczel A. (2016). Curr. Protein Pept. Sci..

[cit4] Farkas V., Ferentzi K., Horváti K., Perczel A. (2021). Org. Process Res. Dev..

[cit5] Rovó P., Stráner P., Láng A., Bartha I., Huszár K., Nyitray L., Perczel A. (2013). Chemistry.

[cit6] Rovó P., Farkas V., Stráner P., Szabó M., Jermendy Á., Hegyi O., Tóth G. K., Perczel A. (2014). Biochemistry.

[cit7] Karageorgos V., Venihaki M., Sakellaris S., Pardalos M., Kontakis G., Matsoukas M.-T., Gravanis A., Margioris A., Liapakis G. (2018). Hormones.

[cit8] Parthier C., Reedtz-Runge S., Rudolph R., Stubbs M. T. (2009). Trends Biochem. Sci..

[cit9] Underwood C. R. , Parthier C. and Reedtz-Runge S., in Vitamins & Hormones, ed. G. Litwack, Academic Press, 2010, vol. 84, pp. 251–278 10.1016/B978-0-12-381517-0.00009-6 21094903

[cit10] Bazarsuren A., Grauschopf U., Wozny M., Reusch D., Hoffmann E., Schaefer W., Panzner S., Rudolph R. (2002). Biophys. Chem..

[cit11] Schröder-Tittmann K., Bosse-Doenecke E., Reedtz-Runge S., Ihling C., Sinz A., Tittmann K., Rudolph R. (2010). Biochemistry.

[cit12] Runge S., Thøgersen H., Madsen K., Lau J., Rudolph R. (2008). J. Biol. Chem..

[cit13] Underwood C. R., Garibay P., Knudsen L. B., Hastrup S., Peters G. H., Rudolph R., Reedtz-Runge S. (2010). J. Biol. Chem..

[cit14] Grace C. R. R., Perrin M. H., Gulyas J., Rivier J. E., Vale W. W., Riek R. (2010). J. Biol. Chem..

[cit15] Pioszak A. A., Parker N. R., Suino-Powell K., Xu H. E. (2008). J. Biol. Chem..

[cit16] Monaghan P., Woznica I., Moza B., Sundberg E. J., Rosenblatt M. (2007). Protein Expression Purif..

[cit17] Wu L., Yujia Z., Lu J., Wang Q., Sun F. (2013). Protein Expression Purif..

[cit18] Lobstein J., Emrich C. A., Jeans C., Faulkner M., Riggs P., Berkmen M. (2012). Microb. Cell Fact..

[cit19] Beckwith J., Prinz W. A., Åslund F., Holmgren A. (1997). J. Biol. Chem..

[cit20] Terpe K. (2003). Appl. Microbiol. Biotechnol..

[cit21] Thapa P., Zhang R.-Y., Menon V., Bingham J.-P. (2014). Molecules.

[cit22] Dawson P., Muir T., Clark-Lewis I., Kent S. (1994). Science.

[cit23] Ollivier N., Dheur J., Mhidia R., Blanpain A., Melnyk O. (2010). Org. Lett..

[cit24] Ollivier N., Vicogne J., Vallin A., Drobecq H., Desmet R., El Mahdi O., Leclercq B., Goormachtigh G., Fafeur V., Melnyk O. (2012). Angew. Chem., Int. Ed..

